# Primary care-based interventions for secondary prevention of opioid dependence in patients with chronic non-cancer pain taking pharmaceutical opioids: a systematic review

**DOI:** 10.3399/BJGPO.2024.0122

**Published:** 2024-11-27

**Authors:** Clare E French, David M Troy, Sarah Dawson, Michael N Dalili, Matthew Hickman, Kyla H Thomas

**Affiliations:** 1 Population Health Sciences, Bristol Medical School, University of Bristol, Bristol, UK; 2 National Institute for Health and Care Research Health Protection Research Unit in Behavioural Science and Evaluation, University of Bristol, Bristol, UK

**Keywords:** chronic pain, prevention, addictions, opioids

## Abstract

**Background:**

Globally, almost one-third of adults with chronic non-cancer pain (CNCP) are prescribed opioids. Prevention of opioid dependence among these patients is a public health priority.

**Aim:**

To synthesise the evidence on the effectiveness of primary care-based interventions for secondary prevention of opioid dependence in patients with CNCP who are taking pharmaceutical opioids.

**Design & setting:**

Systematic review of randomised controlled trials (RCTs) and comparative non-randomised studies of interventions from high-income countries.

**Method:**

We searched five databases for studies on non-tapering secondary prevention interventions, such as tools for predicting dependence, screening tools for early recognition of dependence, monitoring of prescribing or medication, and specialist support. We examined multiple outcomes, including reduction in opioid dosage. Primary analyses were restricted to RCTs with data synthesised using an effect direction plot. Risk of bias was assessed using the Cochrane risk of bias (RoB2) tool.

**Results:**

Of 7102 identified reports, 18 studies were eligible (eight of which were RCTs). Most used multiple interventions or components. Of the seven RCTs at low risk of bias or with ‘some concerns’, five showed a positive intervention effect on at least one relevant outcome, four of which included a nurse care manager and/or other specialist support. The remaining two RCTs showed no positive effect of automated symptom monitoring and optimised analgesic management by a nurse care manager or a physician pain specialist team, or of a mobile opioid management app.

**Conclusion:**

We identify a clear need for further adequately powered high-quality studies. The conclusions that can be drawn on the effectiveness of interventions are limited by the sparsity and inconsistency of available data.

## How this fits in

People with chronic non-cancer pain (CNCP) are often precribed opioids. Preventing opioid dependence among these patients is a public health priority. This review fills a gap in the evidence by providing a comprehensive summary of the effectiveness of primary care-based non-tapering secondary interventions for prevention of opioid dependence in patients with CNCP on pharmaceutical opioids.

## Introduction

CNCP is common, estimated to affect around one-third (range: 8.7%–64.4%) of adults worldwide.^1^ Since the 1990s there has been an upsurge in the number of prescriptions of opioids to manage CNCP, reflecting the challenges of treating the condition without resorting to opioids as well as a complex interplay of social, economic, and healthcare system factors.^2,3^ A systematic review published in 2020 reported that globally just under a third of patients with CNCP are prescribed opioid analgesics.^4^ The harms of long-term opioid use include dependence, misuse, overdose, and death.^5,6^ There is currently no consensus on the most appropriate use of opioids for treating CNCP, with disparities between American, European, and UK guidelines.^7–9^

Widespread prescribing of opioids has resulted in a well-documented and major ongoing public health problem in the US.^10,11^ In the UK, the prescription of opioids in general practice increased by 34% between 1998 and 2016,^12^ and there has since been concerted efforts to reduce prescribing and to prevent patients who are taking opioids from developing dependence.^13^ Interventions to prevent opioid dependence may be categorised into: 1) primary, for example, appropriate pain management and opioid prescribing, using prescribing systems; 2) secondary, for example, early detection of dependence, monitoring of prescribing and/or medication, brief early interventions or referral to treatment, and tapering; and 3) tertiary, for example, pharmacological and non-pharmacological treatment of dependence.^14^

While several reviews have been conducted on secondary prevention interventions for prescription opioid dependence, these have focused on tapering, or more broadly, strategies designed to achieve a reduction or cessation of prescribed opioid use for the management of patients with CNCP.^15–18^ However, other non-tapering secondary prevention interventions have been trialled and/or are currently in use but, to our knowledge, evidence on their effectiveness has not been synthesised. Understanding the effectiveness of these other secondary prevention measures, in addition to tapering, is important for ensuring that primary care practitioners managing patients on prescription opioids are able to make evidence-based decisions to prevent opioid dependence. We conducted a systematic review to address the question ‘What interventions, that could be delivered in the community or primary care, are effective in secondary prevention of opioid dependence in CNCP patients on pharmaceutical opioids?’

## Method

Our review protocol was registered on the PROSPERO international prospective register of systematic reviews: https://www.crd.york.ac.uk/prospero (reference: CRD42022363230).

### Eligibility criteria

Our PICO was as follows:

- *Participants/population*: adults (aged ≥18 years) taking prescribed opioids for CNCP.
- *Intervention:* secondary prevention interventions that could be delivered in the community or primary care, such as those that only used resources that are likely to be available in primary care settings (for example, tools for predicting dependence, screening tools [including urine drug screening] for early recognition of dependence, monitoring of prescribing and/or medications, and brief early interventions or referral to treatment). We excluded studies on tapering interventions alone since there are existing reviews on this.
- *Comparator/control:* any (for example, usual care or other intervention). We only included studies that used a control group.
- *Outcomes*: primary outcomes were early referral to treatment for opioid dependency; reduction in prescribing (for example, reduction in prescribed dosage); and reduction in opioid use (dosage). Secondary outcomes were opioid cessation or opioid free; no illicit use of opioids; improved health-related quality of life; and reduction in opioid-related harms or adverse events (for example, overdose). We only included studies that presented comparative data on ≥1 of these outcomes. We aimed to capture all outcomes that were of clinical relevance and/or of relevance and importance to patients.

We restricted the search to studies from high-income countries and used the UK NHS definition of primary care.^19^ We only included full reports (where only a conference abstract was available and further details could not be obtained from the author, the study was excluded).

### Search strategy

We searched five databases: MEDLINE (Ovid), Embase (Ovid), Cumulative Index to Nursing and Allied Health Literature (CINAHL) (EBSCOhost), Cochrane Central Register of Controlled Trials (CENTRAL), and PsycInfo (Ovid) using a combination of subject headings (MeSH terms), keywords, and search syntax appropriate to each database (see Supplementary Box S1). Each database was searched from inception to 14 November 2022 with no language restrictions. Additionally, we searched ClinicalTrials.gov (https://clinicaltrials.gov) and hand-searched the reference lists of included articles and relevant reviews. Forward citation tracking was done using Web of Science and Google Scholar.

### Screening and data extraction

Search hits were stored in EndNote and independently screened by two reviewers in Covidence (https://www.covidence.org). Discrepancies were resolved through discussion, involving a third reviewer as necessary.

Extracted data were entered into a Microsoft Excel database by one reviewer and checked by a second. Extracted items included study location and setting; study design; participant numbers and demographics; intervention details; follow-up duration; and numerical data on outcomes in each group (for example, mean measurements/values). Where outcome data were reported at multiple follow-up points we used the primary outcome if specified by study authors, or the last follow-up assessment.

### Risk of bias assessment

The Cochrane risk of bias tool (RoB 2) was used for randomised controlled trials^20^ and the ROBINS-I tool (Risk Of Bias In Non-randomised Studies — of Interventions) for non-randomised studies.^21^ Separate assessments were conducted for each outcome that was reported on within a study. Assessments were carried out by two reviewers independently, with discrepancies resolved through discussion.

Before conducting a full assessment of the non-randomised studies we carried out a formal preliminary consideration of bias adapting guidance in the recently published ROBINS-E tool (for reviews of exposures).^22^ For studies judged to be at very high risk of bias based on preliminary considerations a detailed risk of bias assessment was considered unnecessary.

### Data analysis

The primary analysis was restricted to RCTs, with data from non-randomised studies narratively described. Meta-analysis was inappropriate because of the wide variation in intervention types and outcome measures. Instead, we applied SWiM (Synthesis Without Meta-analysis) methods^23^ to synthesise the data more transparently and reproducibly than a traditional narrative synthesis. First, we iteratively developed a classification strategy for intervention types or components and identified which was used in each study. Next, we provided a structured summary of the results from each study, by outcome, presenting both the absolute difference between groups (intervention versus control) in follow-up measurements, and the difference between groups (change in measurement from baseline to follow-up), according to available data.

For RCTs, we used the tabulated results to produce an effect direction plot.^23,24^ Each effect estimate (for example, difference in change from baseline to follow-up between intervention and comparator group) was categorised as showing a positive effect (benefit) or negative effect (harm) based on the observed direction of effect alone. Statistical significance is not considered in the categorisation. Risk of bias was represented by colour coding each study or outcome row in the effect direction plot. Our reporting adheres to both PRISMA^25^ and SWiM^26^ reporting guidelines.

## Results

### Description of included studies

Our searches returned 7102 hits, 18 of which were eligible for inclusion (see Supplementary Figure S1).^27–44^ There were eight trials (four individually randomised^29,31,33,34^ and four cluster randomised^27,28,30,32^), and 10 non-randomised studies.^35–44^ All studies were conducted in the US and published from 2010 onwards. Sample sizes among the RCTs ranged from 40 to 985. Studies were undertaken in a range of settings including primary care clinics, pain clinics, and HIV clinics (see Supplementary Table S1).

### Types of interventions

Most studies used multiple interventions or components. The most common component was specialist support – five studies used a nurse care manager,^27,29,30,32,33^ three used a clinical pharmacist,^33,35,39^ and 10 provided other specialist support (for example, a psychologist or addiction specialist).^29–33,36,38,40,41,43^ Seven studies gave provider training or education^28,30,32,36,38,43,44^ and five provided patient training or education.^28,31,34,37,43^ Six used electronic patient registries, patient reports, or tracked the patient population.^27,30,32,36,38,39^ Other intervention components were medical record review, patient evaluation, or patient reports,^28,32,33,36,37^ assessment of risk of opioid misuse,^31,34,36,37^ developing, reviewing, and updating clinic policies, procedures, or guidelines,^30,38,40,44^ academic detailing,^27,30,44^ urine drug tests,^31,40^ motivational (compliance) counselling or interviewing,^31,41^ decision support tools or skills,^27,34^ patient smartphone apps,^28,34^ and use of diaries,^31^ automated symptom monitoring,^29^ patient navigator,^42^ shared medical appointments,^37^ provider feedback or performance tracking and/or incentives,^43^ and individualised feedback on risk of opioid misuse (see Supplementary Tables S2 and S3).^34^

### Types of outcomes

The most frequently reported outcome was opioid dose (morphine milligram equivalents/morphine-equivalent daily dose [MEDD]),^27–29,32,35,37–44^ followed by Current Opioid Misuse Measure (COMM)^28,30,32–34^ and discontinuation of opioid prescription or opioid cessation;^27,30,32,38,39^ urine drug test result;^31,32^ health-related quality of life;^29,33^ and Drug Misuse Index (DMI),^31^ Opioid Compliance Checklist (OCC),^36^ and Pain Medication Questionnaire.^34^

### Risk of bias

**Figure 1.**
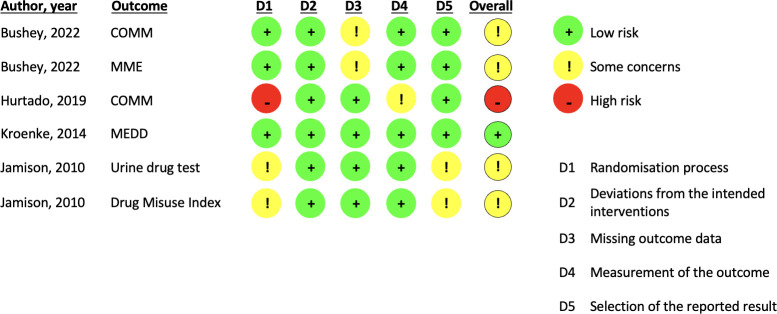
Risk of bias, by study and outcome — randomised controlled trials. COMM = Current Opioid Misuse Measure. MEDD = Morphine Equivalent Daily Dose. MME = (daily) Morphine Milligram Equivalents.

**Figure 2.**
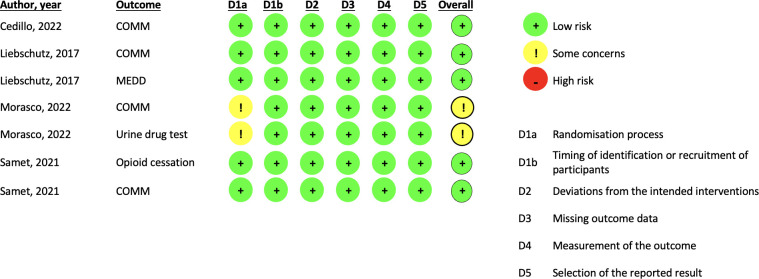
Risk of bias, by study and outcome — cluster randomised controlled trials. COMM = Current Opioid Misuse Measure. MEDD = Morphine Equivalent Daily Dose.

Of the eight RCTs, four were at low risk of bias.^27–30^ Three had some concerns (one because of a lack of information on allocation concealment and lack of a protocol or pre-specified analysis plan;^31^ one because of a lack of allocation concealment;^32^ and one because of a high attrition rate^33^). One RCT was at high risk of bias as a result of concerns around randomisation and allocation concealment (Figures 1 and 2, and Supplementary Tables S4 and S5).^34^

Nine of the 10 non-randomised studies were at critical risk of bias as they did not control for confounding, and there was sufficient potential for confounding that an unadjusted result should not be considered further.^35–40,42–44^ Only the study by Seal *et al* ^41^ was at moderate risk of bias overall.

### Effectiveness of interventions

**Figure 3.**
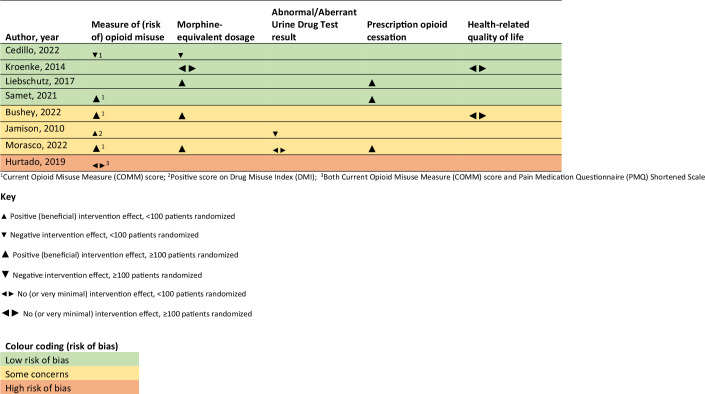
Effect direction plot (randomised controlled trials and cluster randomised controlled trials only).

Data from RCTs are presented in an effect direction plot (Figure 3). Detailed outcome data are provided in Supplementary Tables S6 (RCTs) and S7 (non-randomised studies).

#### Among RCTs at low risk of bias or with some concerns

Five of the seven RCTs at low risk of bias or with some concerns showed a positive effect of their intervention on at least one relevant outcome.^27,30–33^ Four of these used a nurse care manager and/or other specialist support: combination of nurse care manager, academic detailing, decision support tools, and web-based electronic registry;^27^ nurse care manager with other specialist support, an interactive electronic registry, opioid education and academic detailing, and reviewing resources and guidelines;^30^ nurse care manager plus other specialist support;^32^ and nurse care manager-delivered medication management and analgesic optimisation, with input from physician and pharmacist.^33^ Meanwhile, Jamison *et al* used a combination of monthly urine screens, completion of the OCC, education, and motivational compliance counselling.^31^ This was a small study and did not report baseline data for the DMI outcome so should be interpreted with caution.

There was no (or very minimal) effect of automated symptom monitoring and optimised analgesic management by a nurse care manager or physician pain specialist team.^29^ Meanwhile, Cedillo *et al* showed a negative effect of an opioid management app on both COMM and MEDD but this was a small study (*n* = 40).^28^

#### Among non-randomised studies at low or moderate risk of bias

The one non-randomised study at moderate risk of bias showed that an integrated pain team clinic resulted in a greater reduction in opioid dose from baseline than usual primary care.^41^

## Discussion

### Summary

Our review fills an important gap in the synthesis of evidence on the effectiveness of secondary prevention interventions for dependence on prescription opioids. We identified 18 comparative studies, including eight RCTs that used a range of intervention components and outcomes.

We focused our main analyses on RCTs as the strongest source of evidence. Most RCTs used multiple interventions or components and none applied these in a rigorous stepwise fashion. It is therefore difficult to identify the effective components. However, four of the five RCTs (at low risk of bias or with some concerns) that showed a positive intervention effect on at least one relevant outcome included a nurse care manager and/or other specialist support.

Two studies investigated the use of mobile apps,^28,34^ one of which was an RCT at high risk of bias.^34^ Neither demonstrated a beneficial effect of the apps trialled. However, this finding is specific to the apps used. The development and trialling of apps for patient use is an area that may warrant further research.^45^

### Strengths and limitations

We conducted a comprehensive review, searching multiple databases and assessing risk of bias using rigorous tools. While it was not appropriate to meta-analyse the data, rather than simply providing a narrative synthesis we used the SWiM approach to interrogate and synthesise the data more formally and reproducibly. The effect direction plot provides transparent links between the data synthesis and accompanying narrative. However, there are some limitations to this approach, for example, the plot provides no information on the magnitude of effects.

To capture all available evidence we sought both RCTs and non-randomised studies. However, studies on the topic were scarce, and nine of the 10 non-randomised studies were at critical risk of bias. Even among the RCTs, some outcome reporting was sub-optimal and inconsistent across studies. Not all studies clearly reported the difference between groups (intervention versus control) in the change in outcome measurement from baseline to follow-up. We therefore also present the difference between groups in follow-up measurements alone. Such comparisons, where not at least adjusted for baseline measurements, should be interpreted with caution.

The scarcity of high-quality studies, combined with substantial heterogeneity across studies in both intervention components and outcomes, meant it was not possible to identify with certainty specific types of interventions or components that are most effective. Intensity of intervention could play a role but it was not possible to assess its impact on outcomes. Other limitations of available data were the small sample sizes for some studies, and that all studies were from the US, which may limit generalisability — the US has a privatised primary care system compared to the publicly funded systems used in the UK and some other European countries. There are important differences in the structure of the two systems, as well as in the availability of resources.

### Comparison with existing literature

We are not aware of any published reviews on this specific topic. However, there are commonalities with reviews on the broader topic of interventions to prevent dependence on pharmaceutical opioids. Notably, these have reported similar difficulties in drawing conclusions because of the lack of robust evidence. Since our review focused on non-tapering interventions there is very minimal overlap in the studies included in our review and those included in the existing reviews on tapering interventions.

A review on interventions to reduce long-term opioid treatment for CNCP by Avery *et al* included 36 studies. Most were at high risk of bias and the authors noted further difficulties in drawing conclusions because of the small sample sizes and statistical heterogeneity.^16^ Another review by de Kleijn *et al* investigated a range of opioid reduction methods applicable to the primary care setting, including tapering – no eligible RCTs demonstrated a beneficial intervention effect and the authors raised concerns regarding the high risk of bias of included studies and small sample sizes.^18^ A 2017 Cochrane review of interventions for the reduction of prescribed opioid use in patients with CNCP also found little evidence for effectiveness of the methods trialled.^15^ A 2019 systematic review of 22 studies examining the effectiveness of prescription drug monitoring programmes at reducing opioid-related harms found limited evidence for effectiveness (but noted that they remain more broadly valuable in combating opioid dependency).^46^ Similar to our review, the authors noted substantial heterogeneity between studies and deemed meta-analysis inappropriate.

Our synthesis of RCT evidence indicated that nurse care managers or other specialist support may be important. Multidisciplinary teams in primary care have been shown to be effective at chronic pain management^47^ and reducing opioid use disorder.^48,49^ In our review, nurse care managers were noted to undertake roles such as patient reviews and prescription drug monitoring checks. This may be where they exercise a critical role balancing effective pain treatment and patient safety.^50^ A review of interventions delivered by pharmacists in primary care to optimise opioid therapy for patients with CNCP found that four out of the 14 studies included were successful in decreasing opioid dose and five improved patient opioid safety.^51^ A review of pharmacist-led care showed that pharmacists contribute substantially to effective chronic pain management.^52^ Meanwhile, a review of six studies found some evidence that brief psychological interventions were effective in reducing opioid use or related harms.^53^

### Implications for research and/or practice

We provide a comprehensive summary of interventions that have been trialled for secondary prevention of dependence on prescription opioids. Unfortunately, the conclusions that can be drawn on the effectiveness of these interventions are limited by the sparsity and inconsistency of available data. The role of nurse care managers and other specialist support may be important but requires further investigation. Our findings have important implications for future research – we identify a clear need for further adequately powered, high-quality RCTs, as well as other robust study designs.
